# Systemic Health Associations of Apical Periodontitis: A Systematic Review of Observational Studies

**DOI:** 10.7759/cureus.95273

**Published:** 2025-10-23

**Authors:** Balaji Venugopal, Hemamalini Narasimman, Ananthi Mahalingam, R Naren Kishore, Kavitha Jayavel, Vinitha Ganesan, Sriram Kaliamoorthy

**Affiliations:** 1 Department of Dental Surgery, Government Villupuram Medical College and Hospital, The Tamilnadu Dr. M.G.R. Medical University, Villupuram, IND; 2 Department of Oral Pathology and Microbiology, Government Dental College and Hospital, The Tamilnadu Dr. M.G.R. Medical University, Pudukkottai, IND; 3 Department of Dermatology, Government Villupuram Medical College and Hospital, The Tamilnadu Dr. M.G.R. Medical University, Villupuram, IND; 4 Department of Oral and Maxillofacial Surgery, Vishnu Dental College, Bhimavaram, IND; 5 Department of Periodontology, Government Dental College and Hospital, Cuddalore, IND; 6 Central Research Laboratory for Biomedical Research, Vinayaka Mission’s Medical College and Hospital, Vinayaka Mission’s Research Foundation (DU), Karaikal, IND; 7 Department of Dentistry, Vinayaka Mission’s Medical College and Hospital, Vinayaka Mission’s Research Foundation (DU), Karaikal, IND

**Keywords:** apical periodontitis, autoimmune disorders, cardiovascular disease, diabetes mellitus, osteoporosis, pregnancy complications, systemic diseases

## Abstract

Apical periodontitis (AP) is a chronic inflammatory condition of the periapical tissues, often caused by pulp necrosis. While primarily localized in the oral cavity, evidence suggests that AP may contribute to systemic inflammation, influencing conditions such as cardiovascular disease, diabetes, pregnancy complications, osteoporosis, and autoimmune disorders. This review examines the association between AP and various systemic health conditions. A systematic search of PubMed, Scopus, and EMBASE was conducted, focusing on observational studies reporting systemic outcomes in individuals with AP. Data extraction and risk of bias assessment were conducted with evidence quality evaluated using the GRADE framework and a narrative synthesis by disease category. Of the 37 identified studies, 13 were included in the final analysis. Findings showed moderate-certainty evidence linking AP to increased cardiovascular risk, poorer glycemic control in diabetics, adverse pregnancy outcomes (e.g., preeclampsia, low birth weight), higher AP prevalence in osteoporotic patients, and greater persistence of AP in autoimmune disease cohorts. AP was associated with a two to threefold increase in cardiovascular disease and preeclampsia risk, as well as delayed healing in diabetics. AP may contribute to systemic diseases through shared inflammatory pathways, exacerbating conditions such as cardiovascular disease, diabetes, and pregnancy complications. This review emphasizes the importance of including AP in systemic health assessments and suggests that interdisciplinary management is vital for patients with comorbid conditions. Longitudinal studies are necessary to further explore the causal links between AP and these systemic conditions.

## Introduction and background

Apical periodontitis (AP) is a chronic inflammatory condition of the periapical tissues caused by pulp necrosis due to microbial invasion [1]. Although primarily localized in the oral cavity, emerging evidence suggests that AP may contribute to systemic health issues through the release of microbial products such as lipopolysaccharides (LPS) and proinflammatory cytokines such as tumor necrosis factor-α (TNF-α), interleukin (IL)-1β, and IL-6 [1].

**Figure 1:**
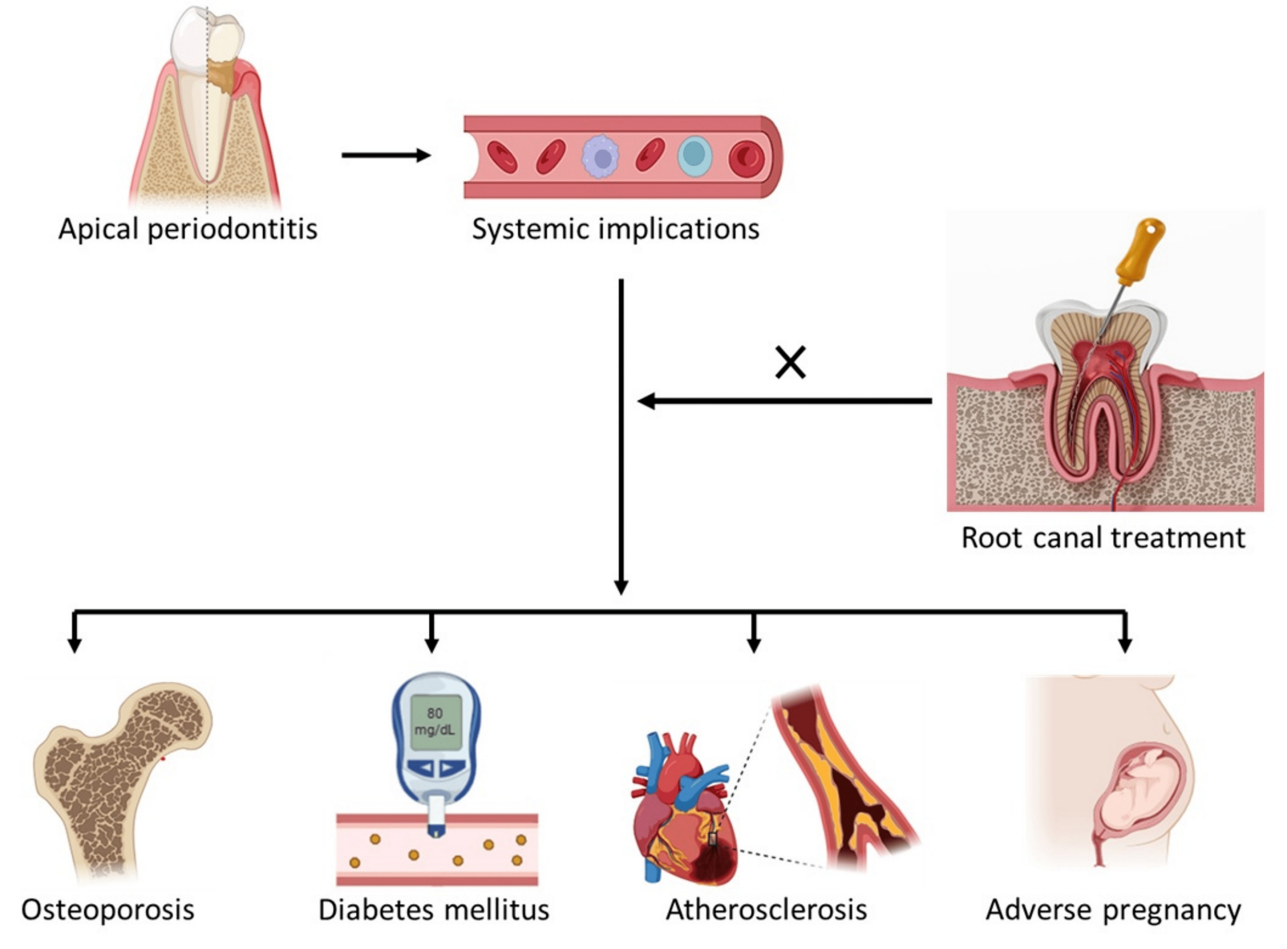
Systemic implications of apical periodontitis: pathophysiological links to cardiovascular, metabolic, and pregnancy-related conditions. Authors’s own image.

The global prevalence of AP, particularly in individuals with untreated necrotic teeth, is well-documented [2]. While AP is often considered a localized issue in dental care, its potential systemic effects are gaining recognition [3]. Studies have indicated that chronic endodontic infections may act as modifiable risk factors for systemic diseases, sharing mechanisms with periodontitis, a more extensively studied condition known for its systemic implications (Figure 1) [4,5].

This review addresses a gap in the literature by systematically evaluating the evidence on the associations between AP and systemic health conditions [6,7]. Previous studies have suggested potential links, but many were narrative reviews or lacked rigorous quality assessments [8,9]. This review aims to critically analyze the literature, using a comprehensive methodology, including risk of bias and evidence quality assessment through the GRADE framework. By doing so, it seeks to clarify the strength of these associations, potentially integrating dental care into broader health management strategies, especially for patients with comorbid systemic conditions.

Research question: Does AP contribute to the development or worsening of systemic diseases, and if so, to what extent does the evidence support these associations?

## Review

Methodology

This systematic review adhered to the Preferred Reporting Items for Systematic Reviews and Meta-Analyses (PRISMA) 2020 guidelines, aiming to synthesize evidence on the associations between chronic AP and systemic health conditions using a rigorous methodology to ensure transparency and minimize bias [10].

A comprehensive search was conducted across PubMed, Scopus, and EMBASE from 1989 to 2025. Search terms combined Medical Subject Headings (MeSH) terms and keywords such as “apical periodontitis,” “endodontic lesions,” “systemic disease,” “cardiovascular,” “diabetes,” “osteoporosis,” “pregnancy outcomes,” and “autoimmune.” The search was limited to studies published in the English language and involving human subjects. A total of 7,588 records were identified, with duplicates removed using EndNote reference management software.

Studies were eligible for inclusion if they met the following criteria: observational studies (cross-sectional, case-control, cohort) involving human subjects with diagnosed AP or root-filled teeth with periapical lesions, and reporting systemic health outcomes such as cardiovascular disease, diabetes, pregnancy complications, osteoporosis, or autoimmune disorders. Exclusion criteria included non-observational studies (e.g., case reports, letters, and experimental studies), studies not focused on human subjects or systemic outcomes related to AP, studies lacking sufficient data or full text availability, and case reports [11].

The selected studies were screened for the titles and abstracts of all identified records for eligibility. Full-text assessment of 37 articles led to the inclusion of 13 studies in the qualitative synthesis, with discrepancies resolved through discussion.

Data extraction was performed using a standardized form to collect study characteristics (authors, year, country, design), population details (sample size, demographics, diagnostic criteria for AP), systemic outcomes (e.g., cardiovascular disease, diabetes, pregnancy outcomes), and key findings (odds ratios (ORs), p-values). Authors were contacted for missing or unclear information.

Risk of bias was assessed using the Newcastle-Ottawa Scale [12,13]. Studies were evaluated based on selection, comparability, and outcome domains, with low, moderate, or high risk of bias assigned. Discrepancies were resolved through discussion.

Evidence quality was assessed using the GRADE framework, which rated the certainty of the evidence as high, moderate, low, or very low based on study design, risk of bias, and other factors [14]. The narrative synthesis provided a comprehensive overview of the associations between AP and systemic diseases.

Results

Study Selection

**Figure 2:**
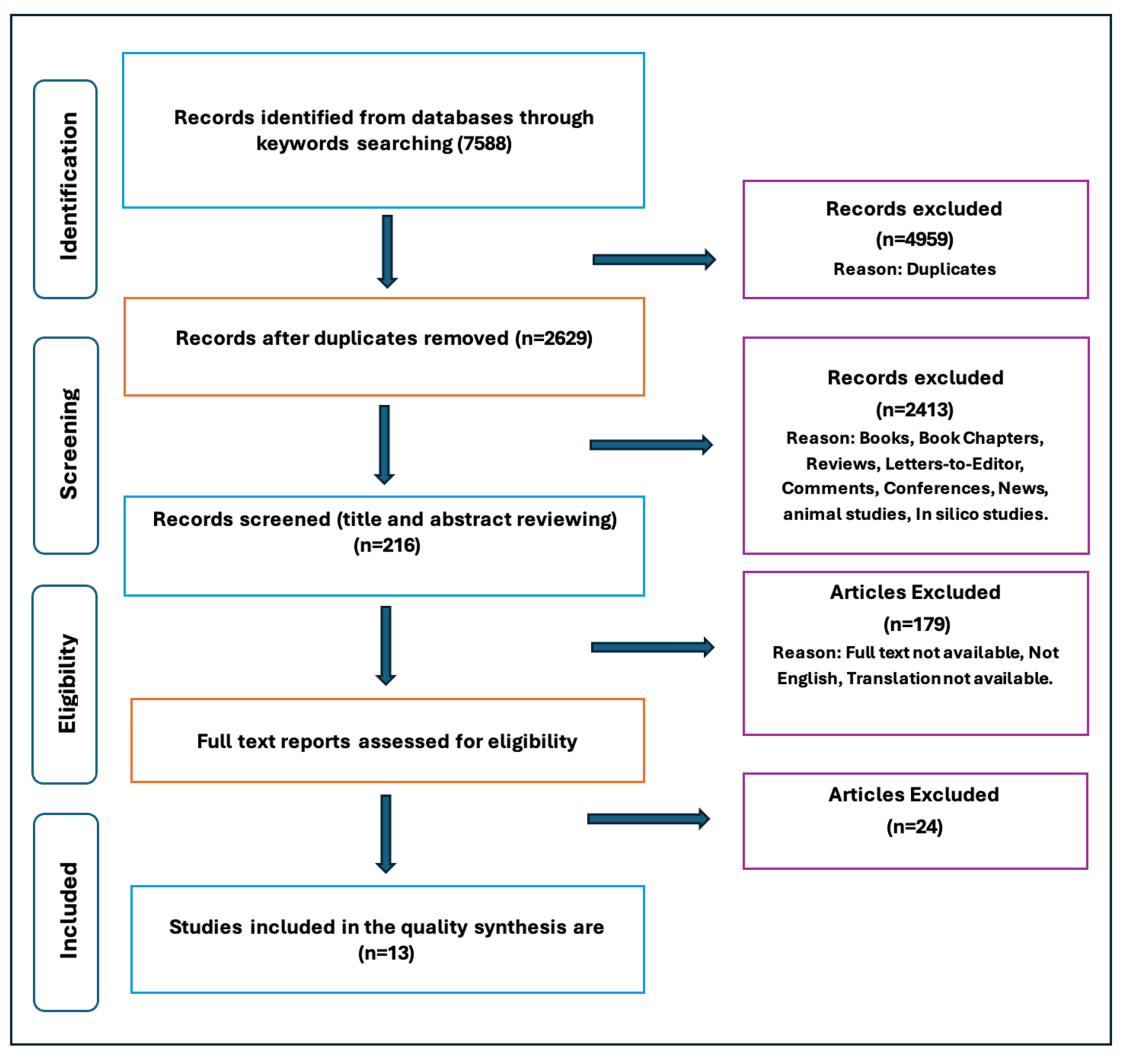
Flowchart illustrating study selection.

The literature search initially identified 7,588 records from PubMed (n = 3,129), Scopus (n = 3,354), and EMBASE (n = 1,105). After removing 4,959 duplicates, 2,629 unique records were screened. Following a full-text assessment of 37 articles, 13 studies met the eligibility criteria and were included in the qualitative synthesis (Figure 2).

Characteristics of Included Studies

The included studies consisted of 13 observational studies focusing on different systemic conditions. Four studies explored cardiovascular outcomes, involving myocardial infarction, hypertension, and coronary artery disease. Four studies examined diabetes, including research on glycemic control and AP prevalence in diabetic patients. Additionally, studies addressed pregnancy, osteoporosis, and autoimmune diseases, focusing on outcomes in postpartum women, bone mineral density, and autoimmune conditions. The studies were conducted across various countries, including Finland, Spain, Brazil, the United States, and others, with sample sizes ranging from 52 to approximately 1.65 million participants (Table 1).

**Table 1:** Characteristics of the studies included in the review on the relationship between apical periodontitis and systemic diseases. AP = apical periodontitis; MI = myocardial infarction; CAD = coronary artery disease; ACS = acute coronary syndrome; IBD = inflammatory bowel disease

Author (year)	Country	Design	Population	Sample size	Systemic outcome	Key findings
Mattila et al. (1989) [15]	Finland	Case-control	Adults with AP vs. without	100 MI cases/102 controls	Cardiovascular	Poor dental health (including AP) is associated with higher MI incidence
Caplan et al. (2010) [16]	Spain	Cross-sectional	Hypertensive vs. normotensive adults	40 hypertensive/51 controls	Cardiovascular	Higher AP prevalence in hypertensive patients
Costa et al. (2014) [17]	Brazil	Cross-sectional	Patients undergoing coronary angiography	103 patients	Cardiovascular	AP is linked to a higher incidence of CAD
Liljestrand et al. (2016) [18]	Finland	Cross-sectional	Middle-aged adults	508 adults	Cardiovascular	AP linked to increased risk of ACS events
Sánchez-Domínguez et al. (2015) [19]	Spain	Cross-sectional	Type diabetic adults	83 diabetic patients	Diabetes	Poor glycemic control is associated with worse periapical status
Smadi (2017) [20]	Jordan	Cross-sectional	Type II diabetic vs. non-diabetic adults	145 diabetics/146 controls	Diabetes	Higher AP prevalence in diabetics, especially in root-filled teeth
Khalighinejad et al. (2017) [21]	USA	Case-control	Pregnant women with AP vs. without	60 cases/60 controls	Pregnancy	AP is an independent risk factor for preeclampsia
Harjunmaa et al. (2015) [22]	Malawi	Cross-sectional	Postpartum women	1,024 women	Pregnancy	Maternal AP is associated with shorter pregnancy duration and lower birth weight
López-López et al. (2015) [23]	Spain	Cross-sectional	Postmenopausal women	120 women	Osteoporosis	Osteoporotic women had lower bone mineral density and higher AP prevalence
Katz and Rotstein (2021) [24]	Israel	Retrospective cohort	Osteoporotic vs. healthy patients	~1.65 million records	Osteoporosis	AP prevalence is higher in osteoporotic patients (1.78%)
Boubaris et al. (2024) [25]	Australia	Cross-sectional	Adults with AP lesions	271 lesions	Osteoporosis	Larger AP lesions correlate with greater bone density reduction
Ideo et al. (2022) [26]	Italy	Retrospective cohort	Autoimmune disease patients on biologics	99 patients/99 controls	Autoimmune	Higher AP prevalence in autoimmune patients on biologics
Piras et al. (2017) [27]	Italy	Cross-sectional	IBD patients vs. controls	52 IBD patients/50 controls	Autoimmune	Higher AP prevalence in IBD patients

Consolidation of Findings Based on Systematic Outcomes

Cardiovascular diseases: A consistent association between AP and cardiovascular diseases, including myocardial infarction (MI), coronary artery disease (CAD), and acute coronary syndrome (ACS), was observed. Several studies reported that AP was linked to higher cardiovascular risk, with Mattila et al. (1989) noting a significant association between poor dental health, including AP, and increased MI incidence (OR = 1.5) [15]. Caplan et al. (2006) found that radiographic AP lesions were linked to a higher risk of coronary heart disease, particularly in individuals under 40 years of age [16]. Costa et al. (2014) demonstrated that chronic AP patients had a 2.8-fold higher odds of angiographically confirmed CAD, while Liljestrand et al. (2016) found a significant association between AP and ACS in middle-aged adults [17,18]. Overall, the evidence quality was rated as moderate, with some studies showing risk of bias related to participant selection and outcome measurement.

Diabetes and glycemic control: Several studies indicated that individuals with diabetes had a higher prevalence of AP, particularly in those with poor glycemic control. Sánchez-Domínguez et al. (2015) reported that diabetic patients with poor glycemic control (HbA1c ≥6.5%) had a 3.8-fold higher risk of AP compared to those with well-controlled diabetes [19]. Smadi (2017) observed a 2.5-fold increased prevalence of AP in diabetics compared to non-diabetic controls [20]. Evidence quality was moderate, with consistent findings but some variability in the diagnostic methods for AP.

Pregnancy outcomes: The association between AP and adverse pregnancy outcomes, such as preeclampsia and low birth weight, was reported in several studies. Khalighinejad et al. (2017) identified AP as an independent risk factor for preeclampsia, while Harjunmaa et al. (2015) found that untreated AP in postpartum women was linked to shorter pregnancy duration and lower birth weight [21,22]. The overall evidence quality was moderate to low, with limited sample sizes and potential biases in some studies.

Osteoporosis and skeletal outcomes: AP was found to be significantly associated with osteoporosis, with osteoporotic patients showing a higher prevalence of AP lesions. López-López et al. (2015) reported that postmenopausal women with osteoporosis had a greater prevalence of AP and lower bone mineral density compared to healthy controls [23]. Katz and Rotstein (2021) noted that osteoporotic patients had an AP prevalence of 1.78%, significantly higher than the general population’s prevalence of 0.52% (OR ≈ 3.4) [24]. Boubaris et al. (2024) observed that larger periapical lesion volumes correlated with greater reductions in bone density, suggesting a potential localized effect of AP on bone health [25]. Evidence quality was moderate, but several studies had a high risk of bias due to study design and sample selection.

Autoimmune and immune-mediated conditions: A notable association between AP and autoimmune diseases was found, with studies reporting a higher prevalence of AP in patients with conditions such as rheumatoid arthritis and inflammatory bowel disease (IBD). Ideo et al. (2022) found that patients with autoimmune diseases receiving biologic therapy had a significantly higher prevalence of AP (OR ≈ 3.75) [26]. Piras et al. (2017) reported that individuals with IBD had a higher incidence of AP, particularly among females [27]. The evidence quality was moderate, although most studies had a high risk of bias, particularly in case reports and studies with small sample sizes.

Risk of Bias

The risk of bias assessment indicated that four studies had a low risk, seven studies had a moderate risk, and two studies had a high risk. Common issues included insufficient control of confounding factors, selection bias, and limitations in outcome measurement. Studies with a moderate or high bias were carefully considered in the synthesis, especially those based on self-reported outcomes or small sample sizes (Table 2).

**Table 2:** Risk of bias assessment for the included studies on apical Periodontitis and its systemic implications. NOS = Newcastle-Ottawa Scale; ROBINS-I = Risk of Bias in Non-randomized Studies of Interventions

Author (year)	Design	Selection (Max 4)	Comparability (Max 2)	Outcome (Max 3)	Risk of bias score and overall risk	Comments
Mattila et al. (1989) [15]	Case-Control	4/4	2/2	2/3	8/9 - Low	Assessed using NOS/ROBINS-I
Caplan et al. (2010) [16]	Cross-sectional	3/4	1/2	2/3	6/9 - Moderate	Assessed using NOS/ROBINS-I
Costa et al. (2014) [17]	Cross-sectional	2/4	1/2	2/3	5/9 - High	Assessed using NOS/ROBINS-I
Liljestrand et al. (2016) [18]	Cross-sectional	4/4	2/2	2/3	8/9 - Low	Assessed using NOS/ROBINS-I
Sánchez-Domínguez et al. (2015) [19]	Cross-sectional	3/4	0/2	1/3	4/9 - High	Assessed using NOS/ROBINS-I
Smadi 2017 [20]	Cross-sectional	3/4	1/2	2/3	6/9 - Moderate	Assessed using NOS/ROBINS-I
Khalighinejad et al. (2017) [21]	Case-control	4/4	2/2	3/3	9/9 - Low	Assessed using NOS/ROBINS-I
Harjunmaa et al. (2015) [22]	Cross-sectional	4/4	2/2	2/3	8/9 - Low	Assessed using NOS/ROBINS-I
López-López et al. (2015) [23]	Cross-sectional	3/4	1/2	2/3	6/9 - Moderate	Assessed using NOS/ROBINS-I
Katz and Rotstein 2021 [24]	Retrospective cohort	4/4	1/2	2/3	7/9 - Moderate	Assessed using NOS/ROBINS-I
Boubaris et al. (2024) [25]	Cross-sectional	2/4	1/2	2/3	5/9 - Moderate	Assessed using NOS/ROBINS-I
Ideo et al. (2022) [26]	Retrospective cohort	2/4	1/2	2/3	5/9 - Moderate	Assessed using NOS/ROBINS-I
Piras et al. (2017) [27]	Cross-sectional	2/4	1/2	2/3	5/9 - Moderate	Assessed using NOS/ROBINS-I

Certainty of Evidence (GRADE)

Using the GRADE framework, the certainty of evidence was rated as moderate for most systemic health outcomes, reflecting the limitations in study design, risk of bias, and heterogeneity in diagnostic methods for AP. Strong evidence was found for the associations between AP and cardiovascular disease and diabetes, while the evidence for autoimmune diseases and pregnancy outcomes was of lower certainty due to study design limitations and sample size constraints.

Discussion

This systematic review consolidates evidence linking AP with various systemic health conditions, including cardiovascular disease, diabetes, pregnancy complications, osteoporosis, and autoimmune diseases. AP, traditionally seen as a localized dental issue, may serve as a modifiable risk factor for these conditions. However, the overall quality of evidence was moderate, with several studies showing a high risk of bias and heterogeneity in study designs.

AP was consistently associated with cardiovascular diseases such as MI, CAD, and ACS [15-18]. The likely mechanism involves systemic inflammation triggered by microbial by-products from AP lesions, which may lead to endothelial dysfunction, a precursor to atherosclerosis. Despite these findings, evidence quality was moderate, as many studies were observational and lacked control for confounding factors.

Similarly, AP was found to be strongly associated with diabetes, particularly in individuals with poor glycemic control. Chronic inflammation in diabetes could exacerbate AP, complicating periapical healing and increasing the risk of endodontic treatment failure [19-22]. However, methodological limitations, such as varying diagnostic criteria for AP, mean the evidence remains moderate.

The review also highlighted significant associations between AP and pregnancy complications, including preeclampsia and low birth weight. The likely mechanisms involve immune system dysregulation and widespread inflammation [21-23]. However, the evidence quality was low to moderate, primarily due to small sample sizes and inconsistent study designs.

AP was also linked to osteoporosis, with osteoporotic patients showing a higher prevalence of AP lesions. Larger periapical lesions were associated with greater reductions in bone mineral density, suggesting a localized effect of AP on bone health [24,25]. The evidence quality was moderate, but variability in diagnostic methods for both AP and bone health limits the ability to establish a clear causal relationship.

In autoimmune diseases, a notable association between AP and conditions such as rheumatoid arthritis and IBD was observed [26,27]. The relationship may involve immune dysregulation and chronic inflammation, but evidence remains of low to moderate certainty due to small sample sizes and biases in many studies.

Limitations

Limitations of this review include the predominance of observational studies, the heterogeneity of study designs, and the lack of longitudinal data. Confounding factors such as lifestyle and comorbidities were often not adequately controlled, further limiting the ability to draw definitive conclusions.

Future research should focus on longitudinal cohort studies with standardized diagnostic criteria for AP and control for confounding variables. Investigating the impact of endodontic treatment on systemic health outcomes could provide valuable insights into the potential for managing AP to reduce systemic disease risks.

## Conclusions

While AP is linked to various systemic conditions, the quality of the evidence remains moderate. Further high-quality studies are needed to establish causal relationships and evaluate the benefits of managing AP in reducing systemic health risks. Interdisciplinary management involving dental and medical professionals is recommended for patients with comorbid conditions.
